# Involvement of IL-18 in the Expansion of Unique Hepatic T Cells with Unconventional Cytokine Profiles during *Schistosoma mansoni* Infection

**DOI:** 10.1371/journal.pone.0096042

**Published:** 2014-05-13

**Authors:** Keishi Adachi, Risa Nakamura, Yoshio Osada, Masachika Senba, Koji Tamada, Shinjiro Hamano

**Affiliations:** 1 Department of Parasitology, Institute of Tropical Medicine (NEKKEN), Nagasaki University, Nagasaki, Japan; 2 Global Center of Excellence Program, Nagasaki University, Nagasaki, Japan; 3 Department of Immunology and Parasitology, The University of Occupational and Environmental Health, Kitakyushu, Japan; 4 Department of Pathology, Institute of Tropical Medicine (NEKKEN), Nagasaki University, Nagasaki, Japan; 5 Department of Immunology and Cell Signaling Analysis, Yamaguchi University Graduate School of Medicine, Ube, Japan; Karolinska Institutet, Sweden

## Abstract

Infection with schistosomes invokes severe fibrotic granulomatous responses in the liver of the host. *Schistosoma mansoni* infection induces dramatic fluctuations in Th1 or Th2 cytokine responses systemically; Th1 reactions are provoked in the early phase, whilst Th2 responses become dominant after oviposition begins. In the liver, various unique immune cells distinct from those of conventional immune competent organs or tissues exist, resulting in a unique immunological environment. Recently, we demonstrated that *S. mansoni* infection induces unique CD4^+^ T cell populations exhibiting unconventional cytokine profiles in the liver of mice during the period between Th1- and Th2-phases, which we term the transition phase. They produce both IFN-γ and IL-4 or both IFN-γ and IL-13 simultaneously. Moreover, T cells secreting triple cytokines IFN-γ, IL-13 and IL-4 were also induced. We term these cells Multiple Cytokine Producing Hepatic T cells (MCPHT cells). During the transition phase, when MCPHT cells increase, IL-18 secretion was up-regulated in the liver and sera. In *S. mansoni*-infected IL-18-deficient mice, expansion of MCPHT cells was curtailed. Thus our data suggest that IL-18 produced during *S. mansoni* infection play a role in the expansion of MCPHT cells.

## Introduction

Th1 and Th2 cells play important roles in the immune response to many infectious diseases and in autoimmune disorders [1]–[6]. Th1 and Th2 cells mutually impede their generation, and Th1- and Th2-related cytokines are not thought to be simultaneously secreted from single helper T cells [7], [8]. However, it was recently reported that IFN-γ-producing Th1 cells inherently possess the capacity to convert their cytokine productivity [9]–[12]. Th1 cells stimulated by IL-18 and antigen acquire the potential to produce several Th2-related cytokines, including IL-13, but not IL-4, as well as IFN-γ. Th1 cells which gain productivity of Th2 cytokines are termed “super Th1 cells” [9]–[11]. Indeed, within the IL-18-induced super Th1 cells, Gata3 and T-bet, which are the crucial transcription factors for the induction of Th2 and Th1 cells, respectively, coexist [9]. Whilst some recent studies demonstrate that one transcription factor, promyelocytic leukemia zinc finger (PLZF), which was originally identified as a partner fused with retinoic acid receptors in acute promyelocytic leukemia [13], is indispensable for the dual secretion of IFN-γ and IL-4 from γδ T cells or NKT cells [14]–[16]. It has been also reported that exogenous PLZF leads to the concomitant production of IFN-γ and IL-4 from single T cells upon TCR stimulation [17]. Since PLZF-transgenic T cells seem to convert their nature from differentiated mature types into ‘innate’ types [17], [18], PLZF might be involved in the plasticity of committed T cells, such as Th1 and Th2 cells.

Very recently, we reported that some conventional CD4^+^ T cells acquire atypical cytokine production capacities, producing combinations of “IFN-γ+IL-13” and “IFN-γ+IL-4”, during *Schistosoma mansoni* infection [19]. Furthermore, some of these unique populations displayed the potential for secreting three cytokines concomitantly. Interestingly, the T cell populations showing these unconventional cytokine profiles accumulated in the liver, but not in the spleen. Here we term these cells “Multiple Cytokine-Producing Hepatic T Cells” (MCPHT cells).

In the liver, unique and organ-specific immune systems, composed of specialized cells such as Kupffer cells, NK cells, or NKT cells, are present, showing an immunological environment unlike that of any other immune competent organs or tissues [20]–[23]. Constitutive exposure of large amounts of both enteric and systemic blood-borne antigens does not induce intense activation of the hepatic immune system, indicating the existence of strict regulation machineries in the liver. Upon the disruption of these regulatory machineries by infection with some pathogens such as the hepatitis B virus, runaway immune reactions are induced in the liver, resulting in fulminant hepatitis [24], [25]. The molecular mechanisms underlying such phenomena remain to be elucidated.

Schistosome infection begins with direct penetration of the host skin by the cercariae. Subsequently, the schistosomes invade blood vessels and reach the hepatic portal vein, where they mature, mate, and produce eggs. Oviposition in *S. mansoni* starts 4–6 weeks after the initial cercarial infection. Approximately 300 eggs a day are laid by one female fluke, and many of them enter the liver via the blood. Antigens derived from both the worms and the eggs accumulate in the liver. Fibrotic granulomatous disorders in the liver are the most significant and serious etiology of *S. mansoni* infection, although chronic inflammatory lesions are sometimes observed in several other organs [26]–[29].

In a *S. mansoni*-infected host, Th1- and Th2-related responses are evoked during different infectious periods. In the early phase (3–5 weeks postinfection, PI), Th1-related responses are induced. As oviposition begins (4.5–6 weeks PI), the Th1 components are gradually down-regulated, and Th2 reactions become dominant in the late phase (8 weeks PI∼) [27]. Several previous reports indicate that the immunological balances between Th1 and Th2 responses in *S. mansoni*-infected hosts have implications for severity of pathology [27], [28], [30]–[32]. Intriguingly, the MCPHT cells that we recently reported were found to expand during the period between Th1- and Th2-dominant phases, which we term the “transition phase”.

Here, we show that IL-18 contributes to the expansion of MCPHT cells that are induced during *S. mansoni* infection. Levels of IL-18 in the liver and sera are elevated during the transition phase of the infection, when a significant expansion of MCPHT cells occurs. IL-18-deficient mice displayed severely impaired expansion of MCPHT cells during *S. mansoni* infection. Therefore, our present studies suggest that IL-18 induced during *S. mansoni* infection play a role for the expansion of MCPHT cells within the liver of the host.

## Materials and Methods

### Mice

Female BALB/c mice (6–10 week-old) were purchased from SLC (Shizuoka, Japan). IL-18-deficient mice [33] (6–10 week-old) were kindly provided by Dr. H. Okamura (Hyogo College of Medicine, Nishinomiya, Japan). All mice were maintained under specific pathogen-free conditions. The mice were anesthetized as previously reported [34] with some modification. Briefly, mice were intraperitoneally injected the combination of medetomidine hydrochloride (0.3 mg/kg) and midazolam (4 mg/kg), kept warmed with heating lump, and their consciousness were monitored. In the experiments with portal perfusion, the mice were euthanized.

### Maintenance of the Parasite Life Cycle and Infection of Mice with *S. mansoni*

Maintenance of *S. mansoni* life cycle was conducted as previously described [35], [36]. Mice were anesthetized and percutaneously infected with 25 or 250 *S. mansoni* cercariae as previously described [37]. Egg burden was observed in the feces and the caudate lobe of the liver by microscopy, and in most cases, began at 4–5 weeks PI (data not shown), as previously reported [27]. In some experiments, the number of eggs per gram liver tissue (EPG) was analyzed.

### Intracellular Cytokine Staining (ICS)

In order to monitor cytokine production, ICS technology was employed [38]. After the loss of consciousness, mice were sacrificed, and portal perfusion was conducted. Hepatic lymphocytes were prepared from mice at indicated weeks after the infection as previously described [39]–[41]. In each group, lymphocytes isolated from 3 or 4 mice were pooled in order to obtain sufficient cell numbers. These were then stimulated with plate-coated anti-mouse CD3 (17A2, BioLegend) and anti-CD28 (E18, BioLegend) in the presence of brefeldin A. Cell surface molecules were stained with PE-Cy7-conjugated anti-CD4 (GK1.5, BioLegend), Allophycocyanin (APC)-conjugated or Alexa Fluor 647 anti-mouse CD218a (IL-18Rα, BG/IL18RA, BioLegend). In some experiments, *i*NKT cells were detected with α-galactosylceramide (αGalCer)/CD1d tetramer (KRN7000, Funakoshi and T-Select CD1d Tetramer, MBL). Fixation and permeabilization of the cells were conducted with 2% formaldehyde and 0.5% saponin, respectively. For the detection of intracellular cytokines, FITC-, PE-, or APC-conjugated, corresponding monoclonal antibodies were used (IL-4; 11B11, IFN-γ; XMG1.2, BioLegend; IL-13; eBio13A, eBioscience). Flowcytometric analysis was conducted with FACSCanto II or FACSVerse (BD Biosciences), and the data were analyzed with FlowJo software (Tree Star, Inc.). Culture medium was RPMI-1640 supplemented with 10% FCS, 100 U/ml penicillin, 100 µg/ml streptomycin, 50 µM of 2-mercaptoethanol and 2 mM L-glutamine.

### Flowcytometric Analysis of PLZF

Flowcytometry was used for the analysis of PLZF. In brief, cell surface markers were stained with fluorochrome-conjugated monoclonal antibodies as mentioned above. Fixation, permeabilization, and staining of PLZF and cytokines were performed with FoxP3/Transcription Factor Staining Buffer Set (eBioscience) according to the manufacturer’s instructions. PE-conjugated monoclonal antibody (9E12, BioLegend) was used for the detection of PLZF.

### Assay for IL-18

Right lobes of livers excised from mice were homogenized using a Beads Crusher µT-12 homogenizer (TAITEC) in the presence of protease inhibitor cocktail (Sigma-Aldrich). Total protein concentration of each homogenate was determined with BCA Protein Assay Reagent (Thermo Fisher Scientific) according to the manufacturer’s instructions. The concentrations of IL-18 in each homogenate or serum sample were measured using a commercially available ELISA kit (MBL), which detects mature and biologically active form (18 kDa), but not the 21 kDa pro-form, of IL-18.

### Statistics

All data are given as the mean values of more than three independent experiments. In EPG and IL-18 experiments, the mean and standard deviation (SD) values were calculated with mice in each experimental group. Significance between the control group and treated group was determined with Mann-Whitney U test. *P* values less than 0.05 were considered significant.

### Ethics Statement

All mouse experiments were conducted according to relevant national and international guidelines, and were approved by the Institutional Animal Care and Use Committee at Nagasaki University, and were conducted under the control of the Law (No. 105) and Notification (No. 6) of the Japanese Government pertaining to the use of experimental animals.

## Results

### *S. mansoni* Infection Induces the Elevation of Serum IL-18 levels during the Transition Phase

**Figure 1:**
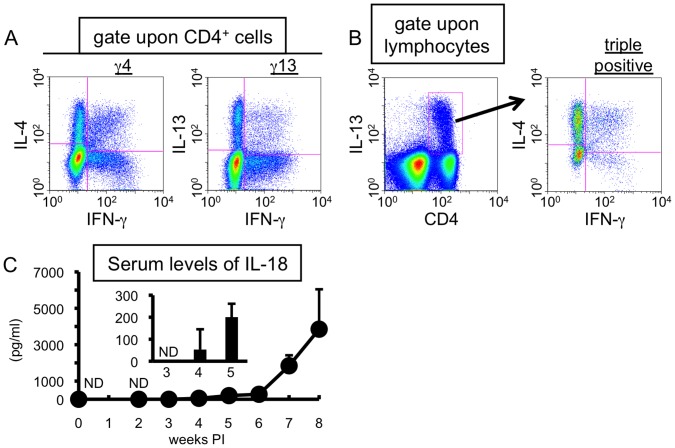
Serum levels of IL-18 are up-regulated during the transition phase of *S. mansoni* infection. (A, B) Hepatic lymphocytes were isolated from *S. mansoni*-infected mice at 6 weeks PI. Cytokine production was analyzed by ICS after TCR stimulation. (C) Sera were obtained at indicted time points, and serum levels of IL-18 were determined by ELISA. Data represent the mean values+SD of three or four mice in each experimental time point. ND, not detected. (A, B) Similar results were obtained in three independent experiments.

Recently we reported that *S. mansoni* infection induced unique CD4^+^ T cells exhibiting unconventional cytokine profiles in the liver of mice during the transition phase (MCPHT cells) [19]. They simultaneously produce Th1- and Th2-cytokines, combinations of “IFN-γ and IL-4” (γ4 cells) and of “IFN-γ and IL-13” (γ13 cells) (Fig. 1A). Furthermore, some of the unique populations acquire the potential for secreting the three cytokines concomitantly (triple positive cells) (Fig. 1B). Meanwhile, it was previously demonstrated that Th1 cells stimulated with antigen and IL-18 produced several Th2-related cytokines, including IL-13, as well as IFN-γ, and these Th2 cytokine-producing Th1 cells were termed “super Th1 cells” [9]–[11]. These data prompted us to investigate the roles of IL-18 in the production of MCPHT cells induced during *S. mansoni* infection. First, we analyzed the kinetics of serum levels of IL-18 after *S. mansoni* infection. As shown in *Figure 1C*, the up-regulation of serum IL-18 levels began after 4 weeks PI. This suggests that the period of serum IL-18 elevation seemed to be synchronized with oviposition (Fig. S1) [27] and the induction of hepatic γ4, γ13, and triple positive cells.

**Figure 2:**
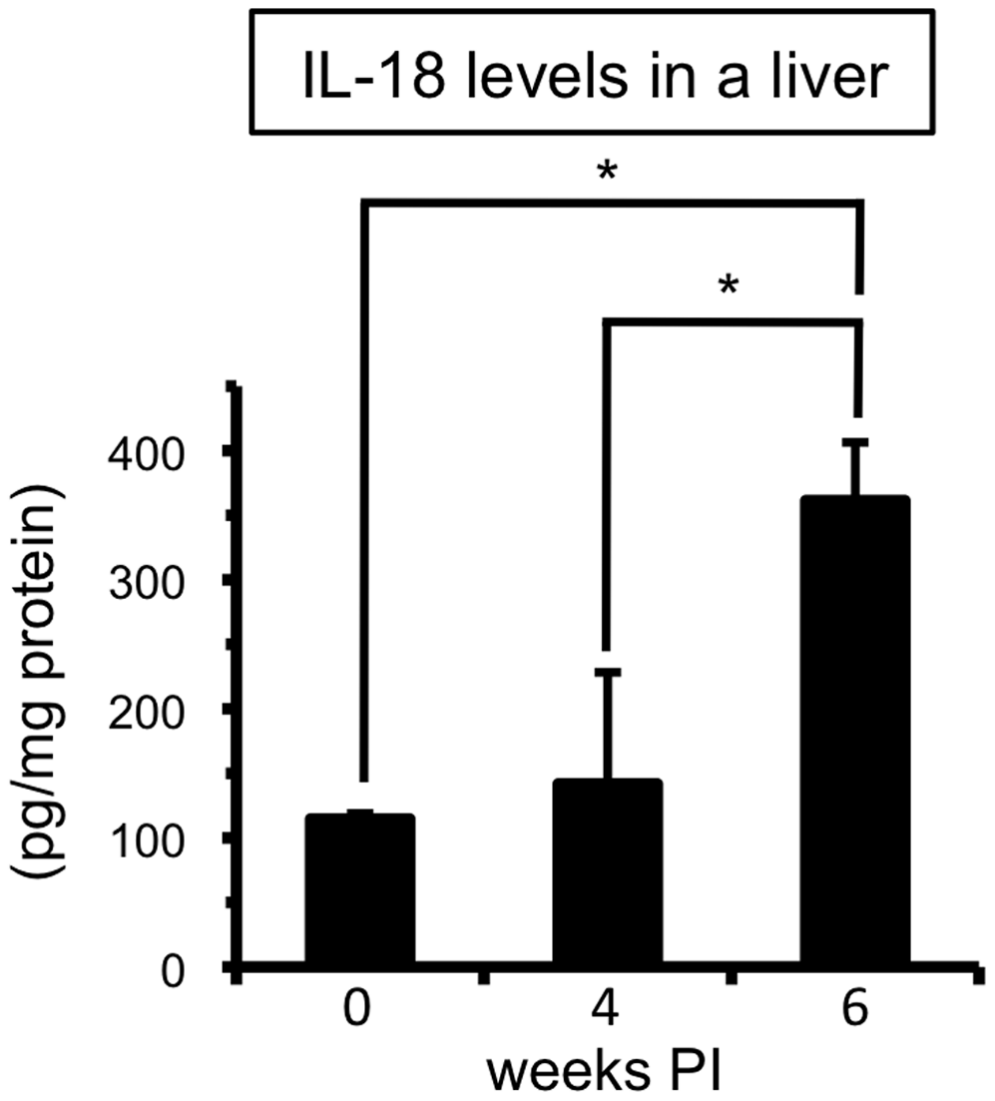
*S. mansoni* infection triggers the increase of IL-18 within a liver. The concentrations of IL-18 in the liver of *S. mansoni*-infected mice were measured as described in the *Materials and Methods*. Data are expressed as mean values+SD of three or four mice in each experimental time point. *0.02<*P*<0.05 (Mann-Whitney U test). Data shown are a representative of three independent experiments.

According to our recent data, accumulation of MCPHT cells occurs in the liver, but not in the spleen during *S. mansoni* infection, indicating that it is a phenomenon specific to the liver [19]. It was demonstrated that Kupffer cells, which are specialized macrophages located in the liver, inherently possess a high potential to produce IL-18 as compared with other primary macrophage lines in some experimental mouse models, such as sequential injection of *Propionibacterium acnes* and LPS [42], [43] and *Plasmodium* infection [39]. Moreover, as *S. mansoni* worms reside and release eggs in the hepatic portal vein [27], [44], pathogenic antigens derived from worms and eggs accumulate in the liver. For these reasons, we next analyzed IL-18 production within the liver during *S. mansoni* infection. As shown in *Figure 2*, IL-18 was detected in the supernatant of liver homogenates isolated from uninfected control mice. This indicates that IL-18 is constitutively produced within the liver, and that the IL-18 produced before the infection might be processed within the liver and/or diluted in the serum to undetectable levels. The levels of IL-18 in the liver were clearly augmented at 6 weeks PI (Fig. 2), suggesting that *S. mansoni* infection elicited the production of IL-18 within the liver, where and when T cells acquire the potential to produce uncommon combinations of cytokines during the infection.

### IL-18 Plays a Role in the Expansion of *MCPHT* Cells during *S. mansoni* Infection

**Figure 3:**
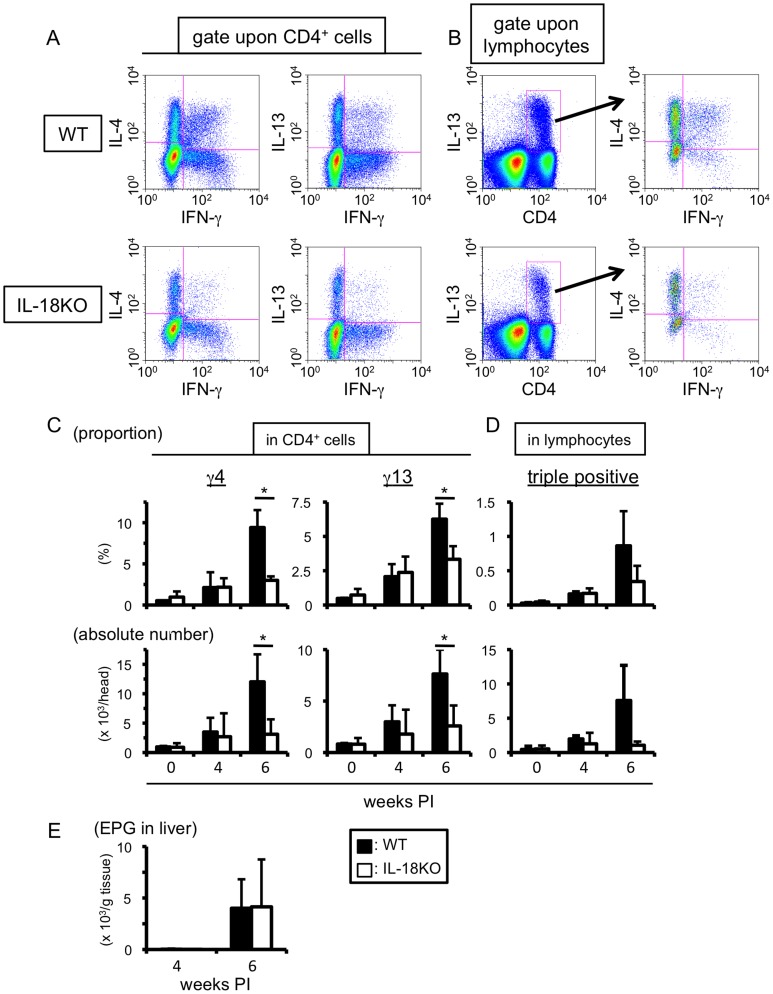
IL-18 plays a role in the expansion of MCPHT cells during *S. mansoni* infection. (A–D) Hepatic lymphocytes were isolated from wild type (WT) or IL-18-deficient (IL-18KO) mice at indicated time points of *S. mansoni* infection, and the proportions and absolute numbers of γ4, γ13 (A and C), or triple positive (B and D) cells were analyzed by ICS upon TCR ligation. (A and B) One example using hepatic lymphocytes prepared at 6 weeks PI was exhibited. (C and D) Upper graphs; the percentages express the proportions in CD4-positive (γ4 or γ13 cells, C) or in lymphocyte (triple positive cells, D) population. Lower graphs; the absolute numbers of γ4, γ13 (C), or triple positive (D) cells were demonstrated. Data are expressed as mean values+SD of three or four mice in each experimental time point. Data shown are a representative of three independent experiments. (C) *0.02<*P*<0.05 (Mann-Whitney U test). (E) EPG of WT and IL-18 KO mice were analyzed at 4 and 6 weeks PI. Data represent the mean values+SD of four mice in each experimental time point. This is one representative of three independent experiments. (C–E) Open bars represent WT mice, and filled bars do IL-18KO mice.

To evaluate the importance of IL-18 for the accumulation of MCPHT cells, we performed experiments with IL-18-deficient mice (IL-18KO). Consistent with our recent report, MCPHT cells were significantly accumulated in the liver of wild type (WT) mice at 6 weeks PI (upper panels of Fig. 3A and B) [19]. Meanwhile, without the infection, very few MCPHT cells were observed in WT and IL-18KO mice (Fig. 3C and D). IL-18KO as well as WT mice displayed some accumulation of γ4, γ13 and triple positive cells in the liver, and no statistical difference was observed between WT and IL-18KO mice at 4 weeks PI, when the increase of MCPHT cell populations began (Fig. 3C and D). This indicates that IL-18 did not play an important role in the initial generation or induction of MCPHT cells. However, in contrast to WT mice (upper panels of Fig. 3A and B), just dull increase and subtle accumulation of MCPHT cells were observed in the livers of IL-18KO mice at 6 weeks PI (lower panels of Fig. 3A and B, and Fig. 3C and D). There were statistical differences in the number and proportion of γ4 and γ13 cells at 6 weeks PI between WT and IL-18KO mice (Fig. 3C). The triple positive cells tended to increase more in WT mice than in IL-18KO mice at 6 weeks PI (Fig. 3B and D). These results suggest that IL-18 is involved in the expansion of MCPHT cell populations. It is noteworthy that the deficiency of IL-18 did not affect the oviposition (Fig. 3E), indicating little difference in the amount of egg antigen flowing into and contained within the liver between WT and IL-18KO mice.

Taken together, these results demonstrate that the endogenous IL-18 induced during the transition phase of *S. mansoni* infection acted as a factor for the expansion of MCPHT cells.

### IL-18 Receptor is Expressed on γ4 and γ13 Cells Induced during *S. mansoni* Infection

**Figure 4:**
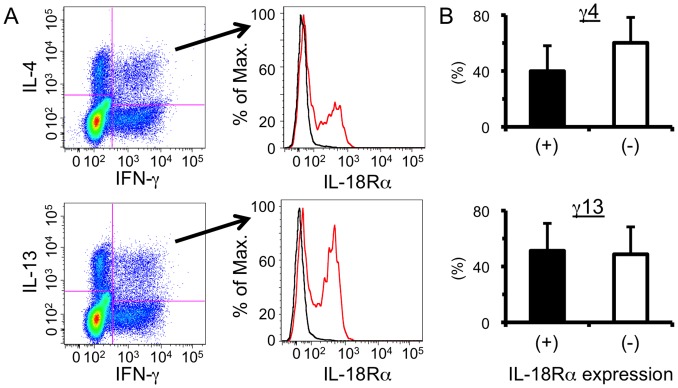
IL-18 receptor is expressed upon some MCPHT cells. (A) Hepatic lymphocytes were isolated from *S. mansoni*-infected mice at 6 weeks PI and ICS was conducted after TCR ligation. The expression levels of IL-18 receptor α (IL-18Rα) upon γ4 (upper panels) or γ13 (lower panels) cells were analyzed. Cells were stained with anti-IL-18Rα (red line) or its respective isotype control (black line). One representative result is shown. (B) The proportions of IL-18Rα-positive (filled bars) or -negative (open bars) population in γ4 (upper graph) or γ13 (lower graph) cells are shown. Data shown are a representative of four independent experiments.

We next analyzed whether IL-18 induced during *S. mansoni* infection was able to directly act upon MCPHT cells through an IL-18 receptor (IL-18R). IL-18R belongs to the IL-1R family and is a heterodimer composed of an IL-18-binding α chain (IL-18Rα, also known as CD218a) and a signaling β chain (IL-18Rβ, initially designated as accessory protein-like, AcPL) [20], [45]–[47]. IL-18R is expressed only at low level upon human and mouse resting T cells, and is upregulated by stimulation with certain cytokines, e.g., IL-12 [48], [49]. In order to investigate the expression levels of IL-18Rα upon hepatic γ4 or γ13 cells accumulated during *S. mansoni* infection, whose expansions were highly influenced by IL-18 (Fig. 3), flowcytometric analysis was conducted. As demonstrated in *Figure 4*, approximately half of the populations of both γ4 and γ13 expressed IL-18Rα. There are also notable differences in IL-18Rα expression levels among the CD4^+^ T cell populations induced in the livers of fluke-infected mice (Fig. S2). As previously reported [45], [50], IFN-γ-singly positive cells, most of which are Th1 cells, showed high-level expression, and in contrast, IL-4- or IL-13-secreting cells, which should be mostly Th2 cells, exhibited low levels of expression. Interestingly, the expression levels of IL-18Rα by γ4 or γ13 cells were intermediate between those of Th1 and Th2 cells (Fig. S2).

Collectively, these results suggest that MCPHT cells expressed IL-18R and that those populations have a potential to react to IL-18 induced during *S. mansoni* infection.

### *i*NKT Cells does not Make a Major Contribution to the Constitution of MCPHT Cells

**Figure 5:**
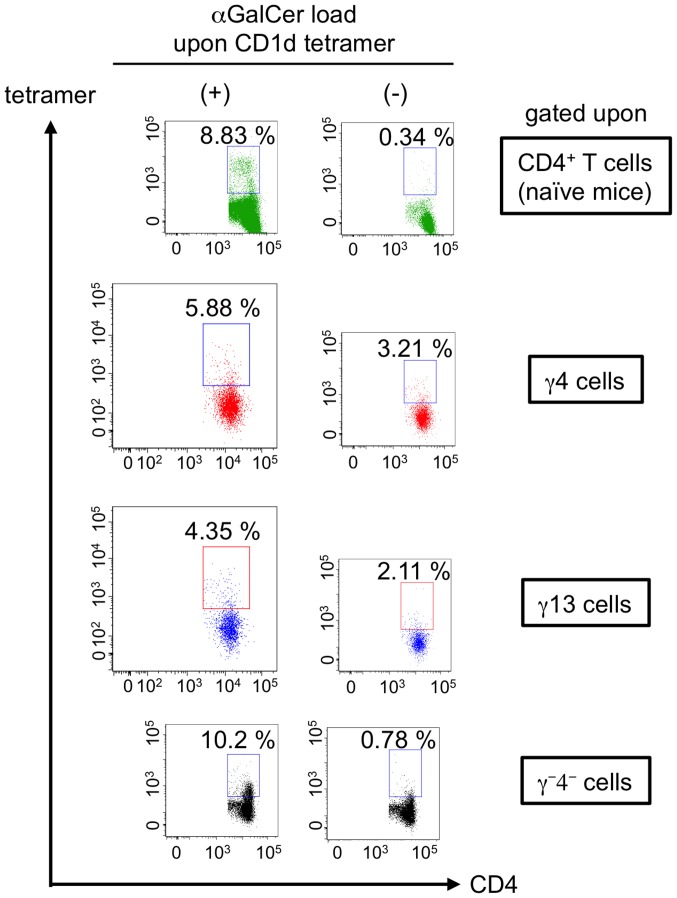
*i*NKT cells are not the major population of MCPHT cells induced during the transition phase. Hepatic lymphocytes were isolated from *S. mansoni*- infected or non-infected, naïve mice and the expressions of *i*NKT cell-specific TCR were analyzed with αGalCer/CD1d tetramer after TCR stimulation at 6 weeks PI. αGalCer-unloaded tetramer was used for the negative controls. The numbers in the insets represent percentages of tetramer-positive populations in CD4^+^ T cells sampled from naïve mice, γ4, γ13, or IFN-γ^−^ IL-4^−^ (γ^−^4^−^) cells from the infected mice. Similar results were obtained in two independent experiments.

We recently demonstrated that some MCPHT cells express DX5, which is also known as integrin α2 and as a mouse pan-NK or NKT cell marker [19]. It has been reported that some *i*NKT cells have capacity to secrete IFN-γ and IL-4 simultaneously [15], [16]. It has been reported that NKT cells are activated in *S. mansoni* infection [51]–[53]. Moreover, it is also shown that *i*NKT cells express IL-18R and produce IL-4 in response to IL-18 [54]. As mentioned above, MCPHT cells contain CD4^+^ T cells expressing IL-18R. These prompted us to investigate whether *i*NKT cells were involved in MCPHT cells induced in the livers of the infected mice. As shown in *Figure 5*, small populations of MCPHT cells were positive for αGalCer/CD1d tetramer although the expression levels of those TCR found to be down-regulated in compared to those of *i*NKT cells sampled form naïve mice probably due to *in vitro* and *in vivo* TCR stimulation. Moreover, the majority of MCPHT cells did not express PLZF, which was reported to play an essential roles in the dual secretion of IFN-γ and IL-4 from *i*NKT cells [15], [16] (Fig. S3).

Taken together, these suggest that most of MCPHT cells induced during the transition phase of *S. mansoni* infection is consisted of the cell population other than *i*NKT cells.

### Antigen Quantity is Important in the Accumulation *MCPHT* Cells Populations during *S. mansoni* Infection

**Figure 6:**
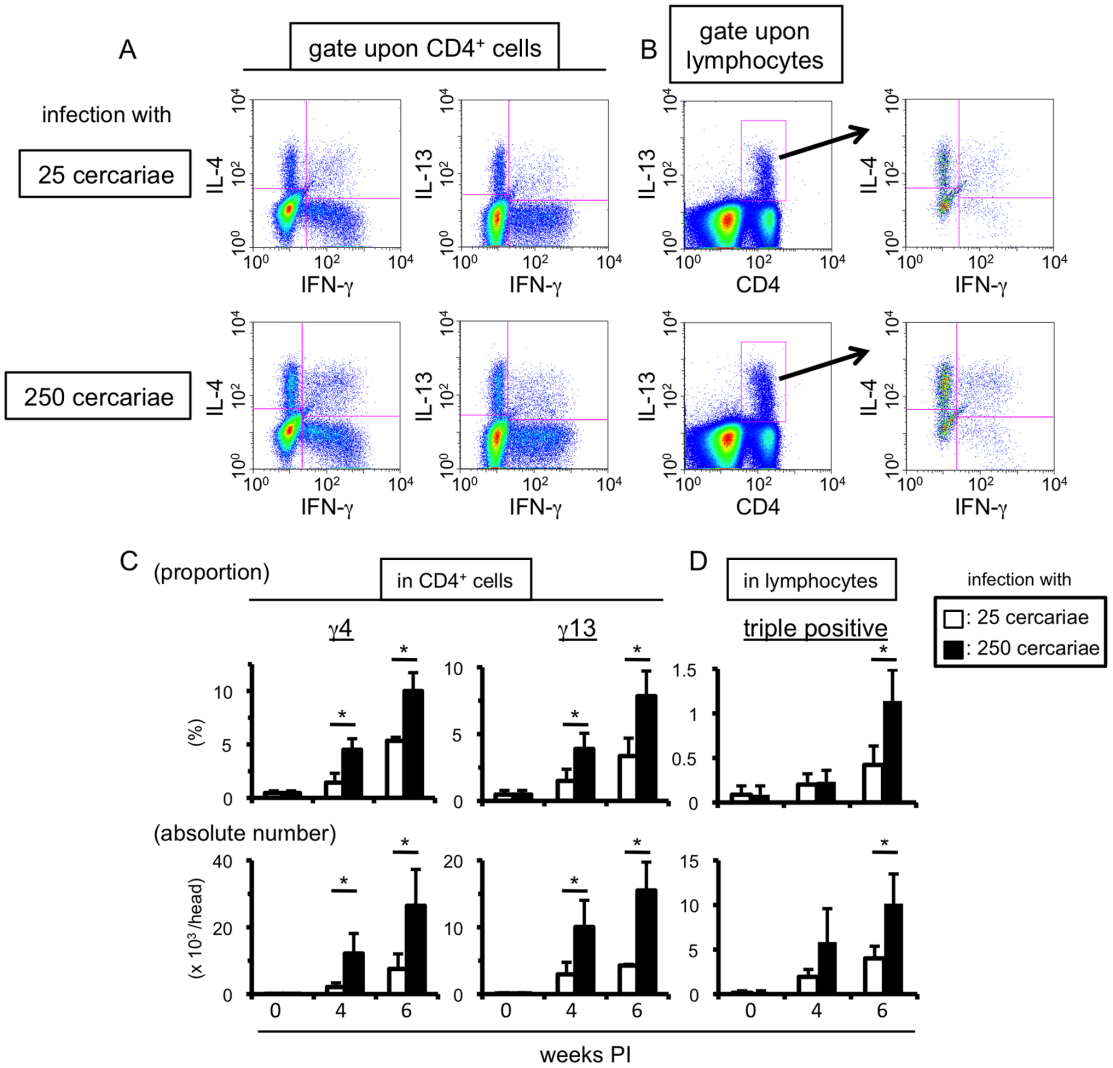
Infection with increased numbers of cercariae augments the induction of MCPHT cells. (A–D) Hepatic lymphocytes were prepared from *S. mansoni*-infected mice at indicated time points, and the proportions and absolute numbers of γ4, γ13 (A and C), or triple positive (B and D) cells were investigated by ICS. (A and B) One example using hepatic lymphocytes prepared at 6 weeks PI was displayed. (C and D) Upper graphs; the proportions in CD4-positive (γ4 or γ13 cells, C) or in lymphocyte (triple positive cells, D) population are shown. Lower graphs; the absolute numbers of γ4, γ13 (C), or triple positive (D) cells are shown. Data are expressed as mean values+SD of three or four mice in each experimental time point. Data shown are a representative of three independent experiments. (C–D) Open bars represent the mice infected with 25 cercariae, and filled bars do those with 250 cercariae. *0.02<*P*<0.05 (Mann-Whitney U test).

As the liver is the primary organ for accumulation of parasitic antigens following the transition phase of *S. mansoni* infection [27], [44] and signaling through TCRs is important for the proliferation of T cells [55], [56], we assessed the influence of the quantities of worm/egg antigens upon the accumulation of γ4, γ13 or triple positive cells in the livers of *S. mansoni*-infected mice. Mice were inoculated with 25 or 250 *S. mansoni* cercariae, and the levels of accumulation of γ4, γ13 or triple positive cells in livers were compared. As expected, mice infected with 250 cercariae exhibited larger EPG values than mice infected with 25 cercariae (Fig. S1). At 6 weeks PI, high proportions and large numbers of the three unique MCPHT cell populations were observed in the livers of mice infected with 250 cercariae, compared with those of mice with 25 cercariae (Figure 6A to D). A more prominent increase in both proportions and in absolute numbers of γ4 and γ13 cells was invoked in the livers of mice infected with 250 cercariae than in those of mice with 25 cercariae even at 4 weeks PI, when oviposition is first initiated and only very small numbers of the eggs are detected in the livers of mice with 25 cercariae (Fig. S1). This data indicates that larger amounts of schistosoma worm/egg antigens stimulated greater accumulation of γ4, γ13 and triple positive cells in the liver during *S. mansoni* infection. Mice infected with 250 cercariae had a greater tendency to show high serum levels of IL-18 as compared with those infected with 25 cercariae, although the differences between those experimental groups were not statistically significant (data not shown).

These results suggest that not only IL-18 induced during the infection but also worm/egg antigens are important for the accumulation of MCPHT cells.

## Discussion

It is thought that Th1- and Th2-responses are incompatible in one immunological circumstance, and that single T cells are ordinarily incapable of producing IFN-γ and IL-4, the most typical Th1- and Th2-related cytokines respectively, simultaneously [7], [8]. However, several previous studies have reported the coexistence of Th1- and Th2-immune conditions in the livers of *S. mansoni*-infected hosts, when symptoms are not severe [27], [28], [30]–[32]. Moreover, we recently demonstrated that *S. mansoni* infection elicited the accumulation of unique CD4^+^ T cell populations exhibiting unconventional cytokine profiles in the liver (MCPHT cells), but not in the spleen, of mice infected during the transition phase, the period between early Th1- and late Th2-superior phases. These hepatic T cell populations produced the combinations of cytokines; IFN-γ+IL-4 and IFN-γ+IL-13. Furthermore, some of the unique populations simultaneously secreted IFN-γ, IL-4, and IL-13. We explored the previously unresolved molecular machineries underlying the accumulation of MCPHT cell populations in the liver during the transition phase of *S. mansoni* infection.

The data presented here suggest that IL-18 induced during *S. mansoni* infection acts as a factor associated with the expansion of MCPHT cells. *S. mansoni* infection stimulated the elevation of IL-18 levels not only in the sera but also in the liver during the transition phase, when the expansion of MCPHT cell populations and oviposition of the trematode begin. IL-18-deficient mice displayed severely impaired expansion of γ4 and γ13 cells in the liver during *S. mansoni* infection. Furthermore, expression of IL-18R was observed in approximately half of both γ4 and γ13 cells.

It is noteworthy that MCPHT cell populations were induced in IL-18KO mice at four weeks PI (Fig. 3). The subsequent increase of these MCPHT cells was not induced in the IL-18KO mice, and this resulted in a considerable reduction in the proportions and the absolute numbers of γ4 and γ13 cells in IL-18KO mice compared to WT control mice at 6 weeks PI (Fig. 3). This suggests that IL-18 is indispensable for the expansion, but not required for the generation, of γ4 and γ13 cells in the liver during *S. mansoni* infection.

The determinant(s) of the generation of MCPHT cells induced following *S. mansoni* infection are unknown. One possible candidate is an adolescent worm product. Indeed, soluble worm antigen preparation (SWAP) has been shown to endow conventional hepatic T cells with the capacity to produce large amounts of IL-4 and IL-13 [37]. SWAP consists of several components including not only T cell antigens but also factors that stimulate, or are targeted by innate immunity. It is probable that SWAP produced by immature and mature worms differs in its composition. Hence, the cytokine profiles of Th1 cells generated during the early phase of *S. mansoni* infection, when the antigens are presented by Kupffer cells affected by immature worm’s SWAP, may be converted into those of the MCPHT cells described here, induced during the transition phase, when the Kupffer cells influenced by mature worm’s SWAP present antigens. As the kinetics of the induction of MCPHT cells and fluke oviposition seem to be identical (Fig. 3 and Fig. S1), the other possible candidate for the determinant(s) of the generation MCPHT cells is soluble egg antigen (SEA), particularly omega-1, which is a glycosylated T2 RNase and most abundantly present in SEA. Omega-1 acts as the major component in SEA responsible for conditioning dendritic cells to convert naïve T cells into Th2 cells [57]–[59]. Therefore, it may be fruitful to investigate the effects of omega-1-immunized hepatic dendritic cells or other antigen-presenting cell populations [60] upon the cytokine productivities of IFN-γ-producing Th1 cells generated within the liver in the early phase of *S. mansoni* infection.

As shown in *Figure S4*, IL-18KO mice infected with 250 cercariae exhibited higher levels of accumulation of MCPHT cell populations than those infected with 25 cercariae. On the basis of the hypothesis concerning the generation of MCPHT cell populations described above, this suggests two possibilities. One possibility is that there may be IL-18-independent and antigen-dependent mechanisms for the expansion of the hepatic γ4, γ13 and triple positive cells, and that large amounts of parasitic antigen could overcome the negative effect of IL-18 deficiency upon expansion of MCPHT cell populations induced following *S. mansoni* infection. In the livers of mice infected with 250 cercariae, larger quantities of SWAP and SEA, both of which might be involved in the generation of MCPHT cell populations, should be accumulated than in those infected with 25 cercariae. The other possibility is, therefore, that the differences in the accumulation of MCPHT cells between the mice infected with 250 and 25 cercariae came from differences in the generation, but not in the expansion, of those cell populations.

As presented in *Figure S2* and reported previously [45], [50], IL-18R was scarcely expressed in IL-4- or IL-13-producing Th2 cells, suggesting that Th2 cells should be little affected by IL-18. Moreover, Th2 cells are mainly generated after seven weeks PI (data not shown), when strong Th2 response-inducing factors, such as SWAP from mature worms and SEA, are abundant in the hepatic environment, and hence, generation of Th2 cells in the liver is accelerated [37], [57]–[59]. For this reason, it is possible that Th2 cells are not the major cellular origin of MCPHT cells that expand following the transition phase of *S. mansoni* infection, although the possibility that Th2 cells change their nature by other unidentified mechanisms cannot be excluded.

In our experiments, histological and serological examination revealed that *S. mansoni* infection induced normal hepatic disorders upon IL-18KO mice (data not shown), suggesting that IL-18 itself plays only a minor role in the etiology of schistosomiasis. However, it remains to be determined whether MCPHT cells play some role in the amelioration or deterioration of disease progression as these cell populations still accumulated to some extent even in IL-18KO mice. In order to make this issue clear, it will be necessary to establish a method to specifically deplete these cells. For this purpose, identification of specific marker(s) or generation machineries for MCPHT cells is required.

We demonstrate that approximately half of the γ4 and γ13 cells expressed IL-18R (Fig. 4 and S2). TCR ligation in the presence of IL-4 down-regulates the expression levels of IL-18R on the T cells [61], [62]. Therefore, the expression levels of IL-18R in MCPHT cell populations described here should be down-regulated by IL-4 in an autocrine and/or paracrine manner. This indicates that the proportions of IL-18R-expressing hepatic γ4 and γ13 cells may be higher at the beginning of their generation than at later periods, and hence, the impact of IL-18 upon MCPHT cells may be the largest at the beginning of their generation and be attenuated as those T cells acquire the capacity to produce IL-4 and expand. Moreover, when IL-4-producing Th2 cells are induced, the down-regulation of IL-18R expression upon those cells might be accelerated. Indeed, both the proportions and absolute numbers of MCPHT cells peaked at 6–7 weeks PI (the end of transition phase), and they gradually decreased after that period despite the existence of IL-18 in the environment (data not shown). This may also suggest that MCPHT cells induced during the transition phase might act as one of the cellular sources of initial IL-4, which triggers Th2 cell generation, and as a ‘bridge’ connecting early Th1 and later Th2 phases of *S. mansoni* infection, which helps the appropriate phase transition within the liver.

Further study will provide us with new tools to prevent and/or to treat hepatic granulomatous disorder induced following *S. mansoni* infection.

## Supporting Information

Figure S1 **Infection with increased numbers of cercariae results in enhanced EPG in the liver.** (Upper panel) EPG in the liver of mice infected with 25 (open bar) or 250 (filled bar) cercariae was analyzed at 4 and 6 weeks PI. (†, Lower panel) The EPG values at 4 weeks PI of mice. Data represent the mean values+SD of three or four mice in each experimental time point. This is one representative of three independent experiments. *0.01<*P*<0.05 (Mann-Whitney U test).
(TIF)

Figure S2 **The expression levels of IL-18 receptor are nonidentical among the hepatic CD4^+^ cell populations.** (Upper panels) Hepatic lymphocytes were isolated from *S. mansoni*-infected mice at 6 weeks PI, and ICS was conducted after TCR stimulation. (Lower panels) The expression levels of IL-18 receptor α (IL-18Rα) upon no cytokine-producing (0, orange line), IL-4-producing (4^+^, black line), γ4 (red line), or IFN-γ-producing (γ^+^, blue line) cells (left panel) or upon no cytokine-producing (0, orange line), IL-13-producing (13^+^, black line), γ13 (red line), or γ^+^ (blue line) cells (right panel) were analyzed. This experiment is representative of four independent experiments.
(TIF)

Figure S3 **PLZF is expressed upon small populations of MCPHT cells accumulated during** ***S. mansoni*** **-infection.** Hepatic lymphocytes were isolated from *S. mansoni*-infected mice at 6 weeks PI and flowcytometric analysis was conducted for the expressions of PLZF after TCR ligation. The right, small insets represent the data using isotype control antibody. The numbers in the insets represent percentages of PLZF populations in γ4 or γ13 cells. Similar results were obtained in two independent experiments.
(TIF)

Figure S4 **High-dose cercariae infection induces some increase of the unique hepatic T cells upon IL-18-deficient mice.** Hepatic lymphocytes were prepared from *S. mansoni*-infected mice at indicated time points, and the proportions and absolute numbers of γ4, γ13 (A), or triple positive (B) cells were investigated by ICS. (Upper graphs) The percentages express the proportions in CD4-positive (γ4 or γ13 cells, A) or in lymphocyte (triple positive cells, B) population. (Lower graphs) The absolute numbers of γ4, γ13 (A), or triple positive cells (B) were displayed. (A and B) Open bars represent the mice infected with 25 cercariae, and filled bars do those with 250 cercariae. Similar results were obtained in two independent experiments.
(TIF)
