# Supplementary figures and images for: Involvement of IL-18 in the Expansion of Unique Hepatic T Cells with Unconventional Cytokine Profiles during Schistosoma mansoni Infection
Source: PLoS One. 2014 May 13;9(5):e96042. doi: 10.1371/journal.pone.0096042 (PMC4019514; doi:10.1371/journal.pone.0096042)

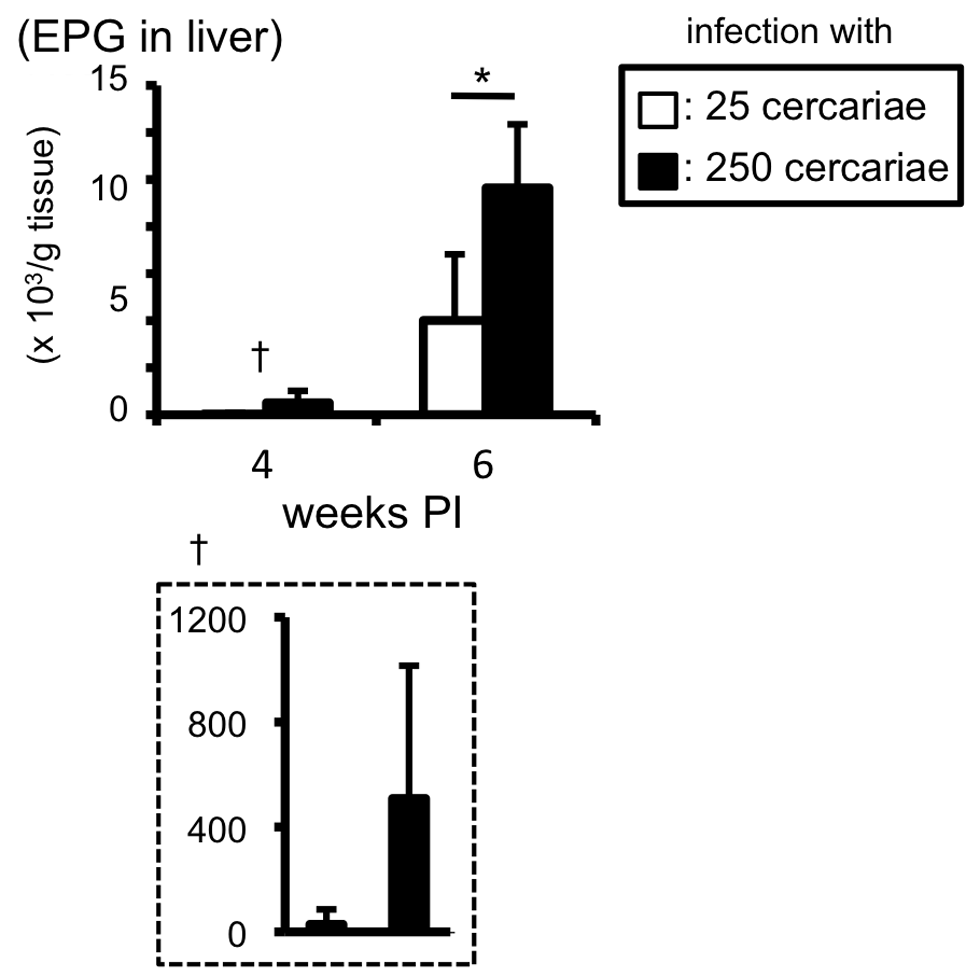

Supplement: Figure S1 — Infection with increased numbers of cercariae results in enhanced EPG in the liver. (Upper panel) EPG in the liver of mice infected with 25 (open bar) or 250 (filled bar) cercariae was analyzed at 4 and 6 weeks PI. (†, Lower panel) The EPG values at 4 weeks PI of mice. Data represent the mean values+SD of three or four mice in each experimental time point. This is one representative of three independent experiments. *0.01<P<0.05 (Mann-Whitney U test). (TIF) [file pone.0096042.s001.tif]

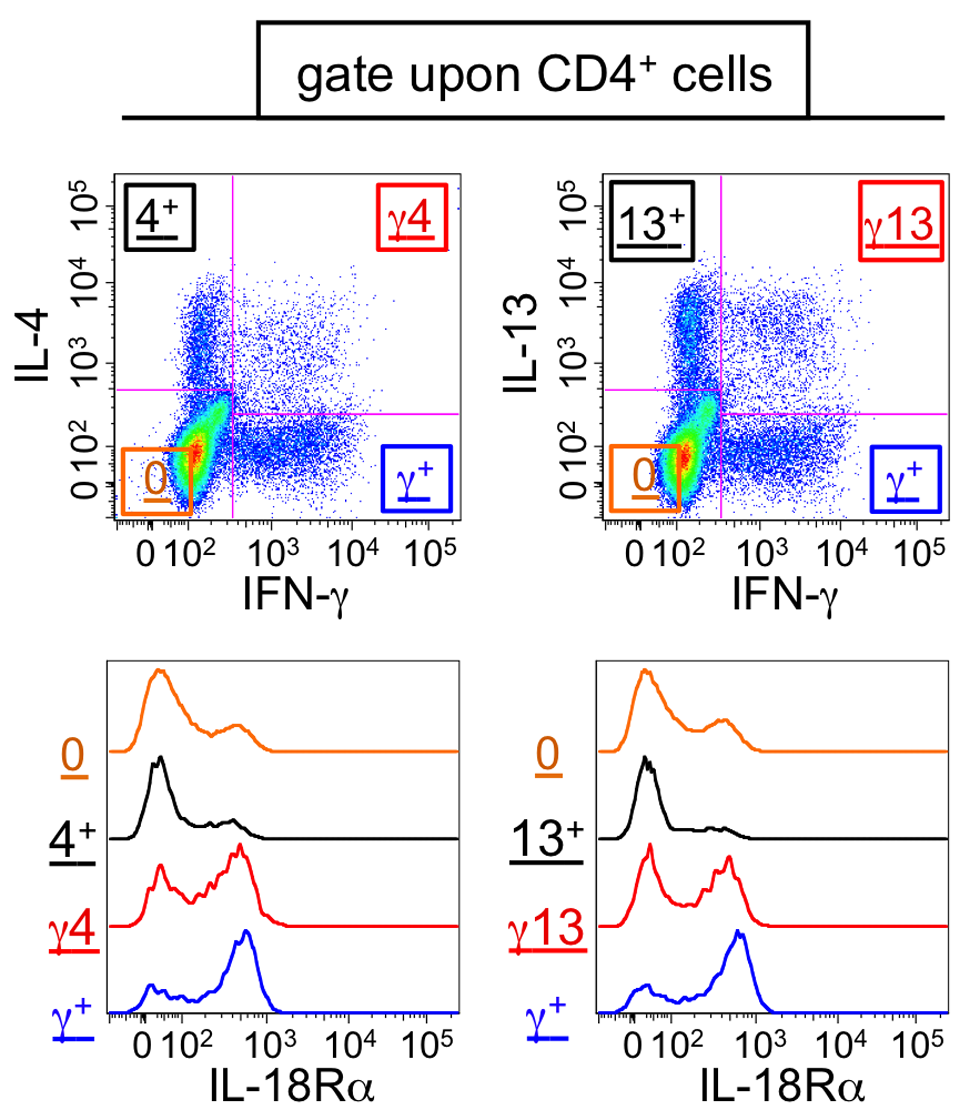

Supplement: Figure S2 — The expression levels of IL-18 receptor are nonidentical among the hepatic CD4+ cell populations. (Upper panels) Hepatic lymphocytes were isolated from S. mansoni-infected mice at 6 weeks PI, and ICS was conducted after TCR stimulation. (Lower panels) The expression levels of IL-18 receptor α (IL-18Rα) upon no cytokine-producing (0, orange line), IL-4-producing (4+, black line), γ4 (red line), or IFN-γ-producing (γ+, blue line) cells (left panel) or upon no cytokine-producing (0, orange line), IL-13-producing (13+, black line), γ13 (red line), or γ+ (blue line) cells (right panel) were analyzed. This experiment is representative of four independent experiments. (TIF) [file pone.0096042.s002.tif]

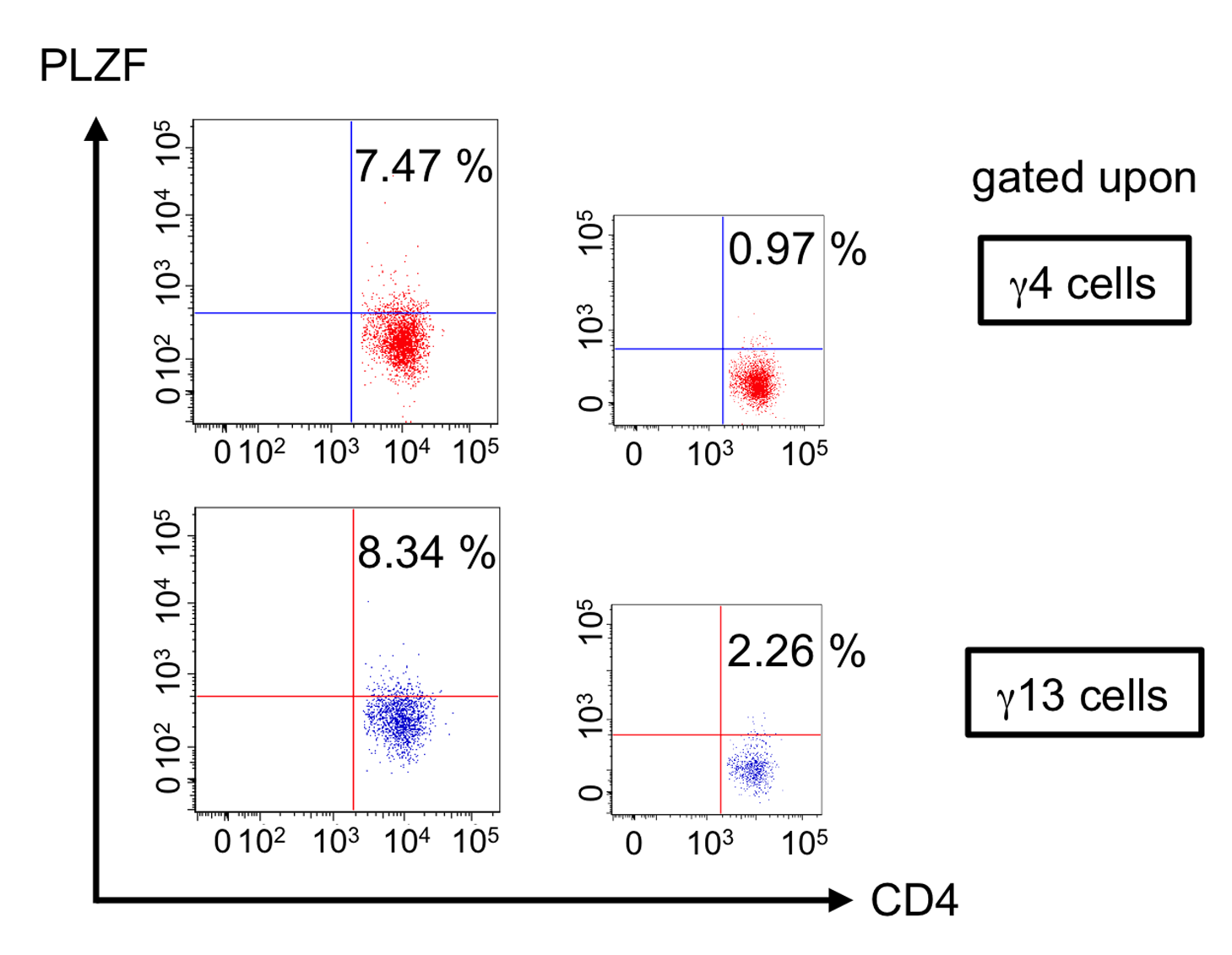

Supplement: Figure S3 — PLZF is expressed upon small populations of MCPHT cells accumulated during S. mansoni -infection. Hepatic lymphocytes were isolated from S. mansoni-infected mice at 6 weeks PI and flowcytometric analysis was conducted for the expressions of PLZF after TCR ligation. The right, small insets represent the data using isotype control antibody. The numbers in the insets represent percentages of PLZF populations in γ4 or γ13 cells. Similar results were obtained in two independent experiments. (TIF) [file pone.0096042.s003.tif]

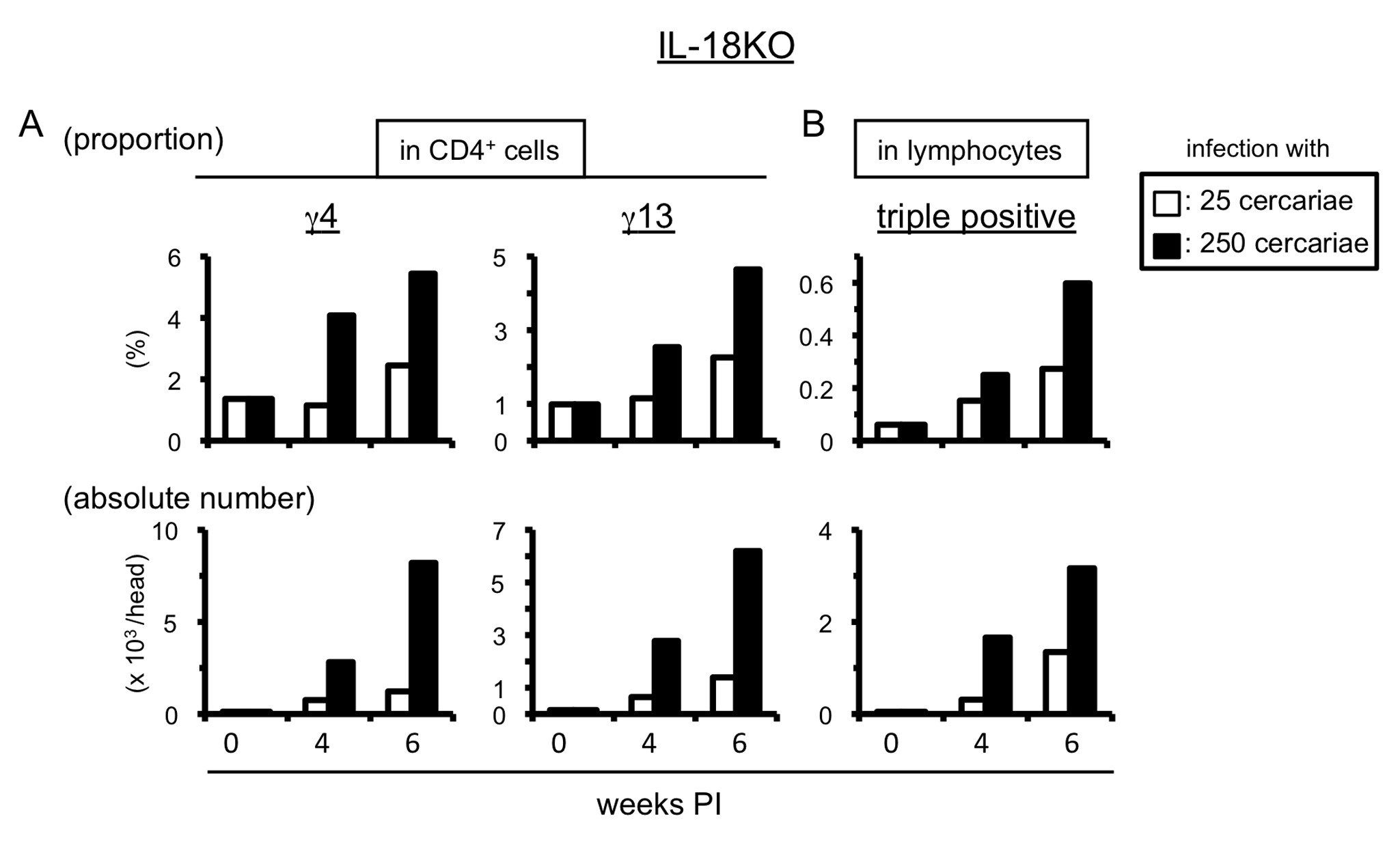

Supplement: Figure S4 — High-dose cercariae infection induces some increase of the unique hepatic T cells upon IL-18-deficient mice. Hepatic lymphocytes were prepared from S. mansoni-infected mice at indicated time points, and the proportions and absolute numbers of γ4, γ13 (A), or triple positive (B) cells were investigated by ICS. (Upper graphs) The percentages express the proportions in CD4-positive (γ4 or γ13 cells, A) or in lymphocyte (triple positive cells, B) population. (Lower graphs) The absolute numbers of γ4, γ13 (A), or triple positive cells (B) were displayed. (A and B) Open bars represent the mice infected with 25 cercariae, and filled bars do those with 250 cercariae. Similar results were obtained in two independent experiments. (TIF) [file pone.0096042.s004.tif]
